# Bioactives and exercise synergize to modulate AMPK and inflammation

**DOI:** 10.3389/fimmu.2025.1670379

**Published:** 2026-01-07

**Authors:** ZhaoHui Sheng, Quan Luo, XianChu Liu, Xingjie Yang

**Affiliations:** 1 School of Physical Education, Hunan University of Arts and Science, Changde, Hunan, China; 2 School of Sports and Arts, Hunan University of Medicine, Huaihua, Hunan, China

**Keywords:** AMPK signaling, natural bioactives, physical activity, immunometabolism, inflammation, mitochondrial biogenesis

## Abstract

AMP-activated protein kinase (AMPK) regulates energy homeostasis and immune responses, making it a key target for immunometabolic disorders like obesity, insulin resistance, and neurodegenerative diseases. This review explores the synergistic effects of plant-derived bioactives (e.g., quercetin, resveratrol, curcumin) and exercise on AMPK signaling, focusing on metabolic and immunological outcomes. A systematic search (PubMed, Scopus, Web of Science, up to June 2025) identified 14 preclinical studies showing that combined interventions enhance AMPK phosphorylation, mitochondrial biogenesis, glucose uptake, and lipid oxidation while reducing pro-inflammatory cytokines (e.g., TNF-α, IL-6) and NF-κB signaling via SIRT1/PGC-1α/Nrf2 pathways. Tissue-specific effects in skeletal muscle, heart, brain, liver, and adipose tissue highlight their therapeutic potential. Variability in study designs and limited clinical data necessitate further mechanistic studies, particularly on epigenetic regulation (e.g., histone acetylation, miRNAs), to optimize personalized non-pharmacological strategies.

## Introduction

Immunometabolic disorders, such as obesity, insulin resistance, cardiovascular diseases, and neurodegenerative conditions, are major global health challenges characterized by dysregulated metabolic pathways and chronic inflammation (1–6). These conditions involve excessive production of pro-inflammatory cytokines (e.g., tumor necrosis factor-alpha (TNF-α), interleukin-6 (IL-6), oxidative stress, and impaired energy homeostasis, leading to tissue dysfunction and disease progression. AMPK, a central regulator of cellular energy balance, orchestrates metabolic and immune responses by promoting mitochondrial biogenesis, fatty acid oxidation, and glucose uptake through GLUT4 translocation, while suppressing inflammatory signaling and oxidative stress (7, 8). As a key therapeutic target, AMPK activation offers a promising approach for managing immunometabolic disorders using accessible, non-pharmacological strategies (9–11).

Disease progression and tissue dysfunction are exacerbated by chronic inflammation, a characteristic of immunometabolic diseases triggered by NF-κB activation and M1 macrophage polarization (12). Since it can inhibit NF-κB and encourage the polarization of anti-inflammatory M2 macrophages, AMPK is a crucial target (13, 14). The limitations of the available pharmacological AMPK activators, such as metformin’s inconsistent efficacy and gastrointestinal side effects (15), underscore the necessity and significance of investigating non-pharmacological approaches. In addressing these constraints, this review highlights the potential of non-pharmacological approaches by summarizing preclinical data on the promising impacts of exercise and plant-derived bioactives on AMPK signaling and immunometabolic outcomes.

Plant-derived bioactives, including polyphenols (e.g., resveratrol, quercetin), curcumin, capsaicin, crocin, saffron, and docosahexaenoic acid (DHA), activate AMPK via direct phosphorylation or upstream regulators such as liver kinase B1 (LKB1) and SIRT1 (16–21). These compounds enhance SIRT1/PGC-1α/Nrf2 signaling, reduce oxidative stress, and modulate cytokine production, yielding metabolic and anti-inflammatory benefits in tissues like skeletal muscle, liver, heart, brain, and adipose tissue. Similarly, physical activity—encompassing endurance, resistance, and high-intensity interval training (HIIT) —upregulates AMPK, improving insulin sensitivity, mitochondrial function, and anti-inflammatory responses (22–24). Preclinical studies from 2014 to 2025 suggest that combining bioactives with exercise synergistically enhances AMPK activation, amplifying downstream effects on glucose metabolism, lipid oxidation, and inflammation compared to either intervention alone (**Table 1**).

**Table 1:** Effects of plant-derived natural products combined with physical activity on AMPK signaling and inflammation.

Natural product	Dosage/duration	Exercise type	Exercise protocol	Model	Key outcomes	Effect type	Ref.
Quercetin	15 mg/kg/day, i.p., 8 weeks	HIIT/MICT	5 sessions/week for 8 weeks (HIIT and MICT separately and in combination with quercetin)	T2DM model	↑ NRF2, ↑ ATGL expression, ↓ PLIN2, ↓ Blood glucose, ↑ Antioxidant capacity	Synergistic	(25)
Grape polyphenols	50 mg/kg/day, oral, 8 weeks	Endurance exercise	Not fully specified	Obese insulin-resistant rats fed high-fat diet	↑ P-Akt/Akt, ↑ P-AMPK/AMPK (EXO, EXOPP); ↓ Fibrosis, ↓ Calcineurin (EXO, EXOPP); ↑ HW/BW ratio (EXO, PP); ↑ SERCA and P-PLB/PLB ratio (PP); ↓ Systolic BP (PP)	No effect	(26)
Grape Polyphenols	Nutritional supplementation during 8 weeks	Endurance training	Treadmill exercise, moderate intensity, 5x/week for 8 weeks	High-fat-diet-induced obese rats (IR)	↑ AMPK activation, ↑ lipid oxidation, ↑ glycogen storage, ↓ insulin resistance, ↑ endurance capacity	Additive	(27)
TCA	30 mg/kg/day, oral, 4 weeks	Aerobic (Treadmill)	Treadmill training (details not given)	MCI mouse model	↑ AMPK/Nrf2, ↑ SIRT1/PGC-1α/Nrf2-ARE; ↑ NQO-1, HO-1, SOD-1; ↓ oxidative stress; improved cognition and memory via antioxidant and mitochondrial signaling pathways	synergistic	(28)
DHA	15% of dietary lipids replaced with DHA; long-term up to 18 months	Treadmill	Moderate treadmill running (duration not specified)	Aged obese female C57BL/6J mice	↓ Steatosis; ↓ lipogenesis genes (Dgat2, Scd1, Srebp1c); ↑ lipid catabolism genes (Hsl, Acox); ↑ AMPK activation, ↑ CPT1A, ↑ PPARα; ↓ inflammatory genes (Mcp1, Il6, TNF-α, TLR4); ↓ ER stress markers; ↑ autophagy-related gene expression	Additive	(29)
Resveratrol	25 mg/kg/day, 4 weeks	Resistance training	Weight-loaded ladder climbing, 3x/week, progressive overload	ICR mice	↑ Grip & anaerobic performance, ↑ glycogen levels, ↓ lactate, ↑ muscle hypertrophy, ↑ aerobic capacity	Synergistic	(30)
Resveratrol	150 mg/kg/day,6 weeks	Aerobic (treadmill)	Moderate treadmill training (details not specified)	Aged SD rats (24 mo	↑ Muscle mass & grip strength, ↑ AMPK & SIRT1, ↓ Acetyl-p53, ↓ Bax/Bcl-2 ratio → anti-apoptotic and muscle-protective effects	Additive	(31)
Resveratrol	10 mg/kg/day, 6 weeks	HIIT, swimming	Swimming HIIT, 5 days/week for 6 weeks, progressive intensity	Aged male Wistar rats (HIIT ± resveratrol)	↑ NAD^+^/NADH, ↑ AMPK, ↑ SOD2 (both HIIT & RES); HIIT ↑ SIRT3, ↓ SIRT4; RES ↓ SIRT3,↑ SIRT4; Combination balanced both pathways	Additive	(32)
Resveratrol	20 mg/kg/day, 8 weeks	Aerobic training	Treadmill running, 6–18 m/min, 5 days/week for 8 weeks	Alzheimer’s rat model (Wistar males)	↑ AMPK, ↑ PGC-1α, ↑ SIRT1; Combination (EX + RSV) significantly more effective than either alone in restoring AMPK/PGC-1α/SIRT1 expression in AD rats	Synergistic	(33)
Capsaicin	0.075% cream (0.6 mg/kg/day) applied 90 min pre-exercise, 6 weeks	Moderate treadmill run	20 min/day, 9–18 m/min, progressive increase every 10 days	Hypoestrogenic obese female rats	↓ Body weight, ↓ abdominal fat, ↓ insulin resistance, ↓ oxidative stress, ↓ pancreatic islet size, ↑ p-AMPK in soleus muscle (only in combination group)	Synergistic	(34)
Capsaicin	4 mg/kg/day orally, 8 weeks	Aerobic training	15–25 m/min, 30–60 min/day, 5 days/week for 8 weeks	Obese Wistar rats on high-fat diet	↑ PGC-1α, ↑ UCP-1 gene expression in VAT; counteracted HFD-induced downregulation; induced browning of WAT via SIRT1/AMPK/PGC-1α/irisin pathway	Additive	(35)
Curcumin	50 or 100 mg/kg/day IP,28 days	Endurance training (eTR)	Treadmill running (specific speed/intensity not detailed)	Healthy Wistar rats	↑ AMPK phosphorylation, ↑ NAD^+^/NADH ratio, ↑ SIRT1, ↑ PGC-1α deacetylation, ↑ OXPHOS subunits, ↑ mitochondrial DNA copy number, ↑ citrate synthase activity, ↑ cAMP/PKA/CREB signaling	Additive	(36)
Curcumin	150 mg/kg/day,8 weeks	Resistance Training	3 sessions/week, climbing with 20–50% body weight load	Obese rats	↓ mTOR, S6K, 4EBP, COL1, COL3, AngII; ↑ AMPK gene expression	Synergistic	(37)
Saffron	40 mg/kg/day, 6 weeks	Resistance Training	Not specified (standard resistance protocol in diabetic rats)	Diabetic rats + *in vitro*	↑ GLUT4, ↑ AMPKα expression; ↓ glucose, TG, cholesterol, VLDL, LDL, HbA1c, HOMA-IR	Additive	(38)
Crocin	25 mg/kg/day, 8 weeks	HIIT & LICT (separate groups)	HIIT & LICT: 5 sessions/week, 8 weeks (details not fully specified)	Diabetic rats	Diabetes ↓ AMPK & NRF1 expression; Crocin + training ↑ AMPK & NRF1 gene expression	Additive	(39)

Despite these promising findings, the synergistic effects of natural bioactives and physical activity on AMPK signaling remain underexplored, particularly in immunometabolic contexts. Variability in experimental designs, including differences in bioactive dosages, exercise regimens, and preclinical models, hinders comprehensive insights. Furthermore, the mechanisms underlying combined interventions—such as their impact on cytokine regulation, redox balance, and epigenetic modifications (e.g., histone acetylation, miRNA expression)—are not fully elucidated. For instance, bioactives and exercise may modulate epigenetic pathways that regulate inflammatory gene expression, offering novel therapeutic avenues. Understanding these interactions could enhance the development of integrated, non-pharmacological strategies for immunometabolic disorders.

This narrative review synthesizes preclinical evidence on the synergistic and additive effects of plant-derived bioactives and physical activity on AMPK signaling, emphasizing tissue-specific responses and immunometabolic pathways. By addressing knowledge gaps, such as optimal dosing, exercise protocols, and epigenetic mechanisms, this review highlights the potential of combined interventions to improve metabolic and inflammatory outcomes. It also outlines future directions for mechanistic and translational research to optimize bioactive-exercise combinations, develop personalized therapies, and advance non-pharmacological approaches for managing immunometabolic disorders, aligning with global health priorities for accessible, cost-effective interventions.

## Methodology and literature search strategy

A systematic literature search was conducted in PubMed, Scopus, and Web of Science from January 2000 to April 2025 to capture studies on AMPK signaling, natural bioactives, and exercise in immunometabolic disorders. The focus on 2014–2024 studies reflects the emergence of relevant preclinical data during this period. Keywords included “AMPK,” “phytochemicals,” “exercise,” “immunometabolism,” and specific bioactives (e.g., “resveratrol,” “curcumin”). Boolean operators (AND, OR) refined the search, restricted to English-language, peer-reviewed preclinical studies. Of 250 studies screened, 14 met inclusion criteria, excluding human studies due to limited clinical data on combined interventions and non-relevant or non-English articles. This approach ensured a focused synthesis of mechanistic insights from animal models.

## AMPK signaling pathway and its role in cellular metabolism regulation

AMPK, a critical enzyme in cellular energy metabolism, acts as the body’s key’ energy sensor’ (40). When cellular energy levels drop, typically indicated by a decrease in ATP (adenosine triphosphate) and a corresponding increase in AMP (adenosine monophosphate), AMPK becomes activated (41). This activation, achieved through phosphorylation at the threonine 172 site on the α-subunit of the AMPK complex, is facilitated by upstream kinases such as LKB1 and calcium/calmodulin-dependent protein kinase kinase 2 (CaMKK2). AMPK, a heterotrimer comprising α (catalytic), β, and γ (regulatory) subunits (42, 43), then shifts cellular activity towards energy-producing processes while efficiently inhibiting energy-consuming activities such as fatty acid, cholesterol, and protein synthesis. This efficient inhibition allows cells to restore energy balance during stress conditions like exercise, fasting, or metabolic disease (41). AMPK’s downstream targets, including PGC-1α, acetyl-CoA carboxylase (ACC), and the mechanistic target of rapamycin (mTOR) (44), further contribute to this efficient restoration of energy balance. Activation of PGC-1α enhances mitochondrial biogenesis, thereby increasing the cell’s ability to produce ATP through oxidative phosphorylation (45, 46). Inhibition of ACC reduces malonyl-CoA production, a molecule that suppresses fatty acid transport into mitochondria, thereby promoting fat oxidation (47, 48). Meanwhile, suppression of mTOR helps conserve energy by reducing cellular growth and protein synthesis during energy shortage (49, 50). Through these efficient mechanisms, AMPK emerges as a central regulator of energy homeostasis, particularly in tissues with high energy demands such as skeletal muscle and cardiac tissue.

## AMPK and immune regulation

Beyond its metabolic roles, AMPK modulates immune responses critical to immunometabolic disorders. By inhibiting NF-κB signaling, AMPK reduces pro-inflammatory cytokine production (e.g., TNF-α, IL-6), as seen in obesity and diabetes models (13, 19). Additionally, AMPK promotes M2 macrophage polarization (51), enhancing anti-inflammatory responses via IL-10 and arginase-1 expression (52). These effects, mediated through SIRT1 and PGC-1α (53), mitigate chronic inflammation in tissues like adipose and liver, positioning AMPK as a key integrator of metabolic and immune homeostasis.

## Exercise and its effects on AMPK signaling

Exercise, particularly aerobic activities like running, swimming, or cycling, is a potent physiological stimulus for AMPK activation (22, 54). As ATP is rapidly consumed in muscle cells, leading to a rise in AMP and activation of AMPK, we can appreciate the numerous metabolic benefits (55). AMPK’s role in promoting fatty acid oxidation by inhibiting ACC, thereby facilitating the transport of fatty acid into the mitochondria, and enhancing glucose uptake by promoting the translocation of GLUT4 to the muscle cell membrane (56, 57) is crucial. Understanding these metabolic benefits is key to our fitness journey.

Exercise-induced AMPK activation is a transformative process that inspires us to push our limits and achieve our fitness goals. It significantly enhances our endurance, a testament to the power of AMPK activation in encouraging us to strive for better fitness levels (22). This process increases PGC-1α expression, leading to enhanced mitochondrial content and function. Both HIIT and moderate-intensity continuous training (MICT) can activate AMPK, with HIIT potentially triggering a stronger response due to its greater energy demands in a shorter time frame (22).

## Natural polyphenols and their effects on AMPK activation

Natural polyphenolic substances have significantly impacted AMPK activity (11, 58, 59). They can do so directly by altering the AMP/ATP ratio, a key indicator of cellular energy status, or indirectly through their anti-inflammatory and antioxidant properties (11).

Resveratrol, quercetin, and EGCG have all been extensively studied in this context. For instance, quercetin, a flavonoid in apples, onions, and berries, can increase AMPK phosphorylation. In this process, AMPK is activated by adding a phosphate group, boosting PGC-1α production, particularly in heart and muscle tissues (11, 60, 61). Quercetin also lowers blood sugar, promotes mitochondrial function, and enhances fat oxidation (62). Similarly, resveratrol found in red wine and grapes increases the NAD^+^/NADH ratio, activates AMPK, activates SIRT1, and stimulates PGC-1α, leading to improved mitochondrial function, reduced inflammation, and increased insulin sensitivity (63–70). EGCG, the primary catechin in green tea, modulates mitochondrial energy status and oxidative stress to activate AMPK, thereby supporting metabolic benefits such as increased glucose tolerance and fat reduction (71–73).

The discoveries in metabolic health and nutrition are of utmost importance. By encouraging ATP-generating processes and suppressing energy-intensive ones, AMPK plays a critical and enlightening role in controlling energy metabolism. AMPK, or adenosine monophosphate-activated protein kinase, is a key regulator of cellular energy balance. It is often referred to as a ‘metabolic master switch’ because it helps to coordinate the body’s response to changes in energy levels. Exercise is a potent and natural AMPK activator that enhances body composition, mitochondrial health, and metabolic efficiency (54). Meanwhile, polyphenol-rich substances like EGCG, resveratrol, and quercetin provide extra ways to control AMPK activation, especially when paired with physical activity. The prevention and treatment of metabolic diseases like obesity, T2D, and cardiovascular disease will be significantly impacted by these discoveries.

## Converging pathways, amplified outcomes: bioactives and exercise co-activate AMPK signaling in preclinical models

The integration of plant-derived bioactives and physical activity offers a potent non-pharmacological strategy for managing immunometabolic disorders through AMPK signaling. Preclinical studies from 2014 to 2024 demonstrate that combining bioactives (e.g., quercetin, resveratrol, curcumin, capsaicin, crocin, saffron, DHA) with exercise modalities (HIIT, endurance, resistance training) enhances AMPK activation, promoting metabolic remodeling and suppressing inflammation across tissues such as skeletal muscle, heart, liver, brain, and adipose tissue. These findings align with the immunometabolic framework, where AMPK regulates energy homeostasis, mitochondrial biogenesis, glucose uptake, lipid oxidation, and inflammatory pathways, offering therapeutic potential for obesity, insulin resistance, type 2 diabetes, cardiovascular diseases, neurodegenerative conditions, NAFLD, and sarcopenia. This section synthesizes key findings from 14 preclinical studies, interprets their immunometabolic implications, and highlights limitations and future research directions to advance personalized therapeutic strategies.

## Synergistic and additive effects on AMPK signaling

**Figure 1:**
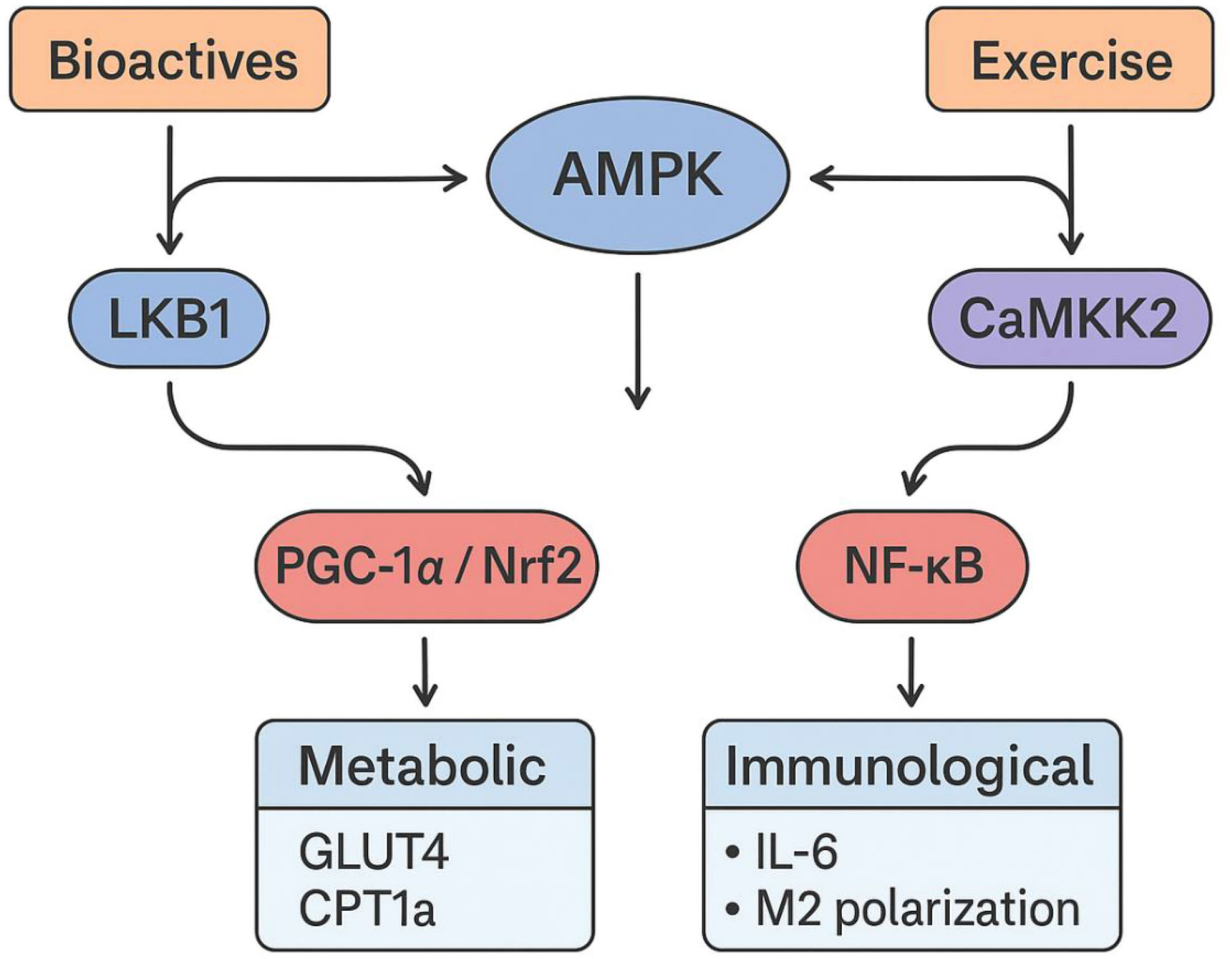
AMPK activation by bioactives (via LKB1/SIRT1) and exercise (via CaMKK2), converging on the PGC-1α/Nrf2 and NF-κB pathways, with tissue-specific metabolic (e.g., GLUT4, CPT1A) and immunological (e.g., IL-6, M2 polarization) outcomes.

The synergistic and additive effects of natural bioactives and physical activity on AMPK signaling are evident across diverse preclinical models, highlighting their potential to enhance metabolic and immune outcomes (**Figure 1**). Khajehlandi et al. (25) demonstrated that quercetin supplementation (15 mg/kg/day) combined with HIIT or MICT in diabetic rats significantly increased nuclear factor erythroid 2-related factor 2 (NRF2) and adipose triglyceride lipase (ATGL) expression while reducing perilipin 2 (PLIN2) and blood glucose levels. Both HIIT and MICT were performed on a treadmill for 8 weeks, five sessions per week, showing that exercise modality influences the extent of molecular activation. These adaptations indicate improved cardiac lipolysis, reduced lipid accumulation, and enhanced antioxidant defense, mitigating inflammation-driven lipotoxicity in the diabetic heart. Similarly, Kan et al. (28) reported statistically significant synergistic effects (confirmed by two-way ANOVA) of resveratrol (25 mg/kg/day) and resistance training in mice, which involved ladder climbing with progressive loads (three sets of 8–10 repetitions, five days per week for 8 weeks). This combined intervention improved anaerobic capacity, grip strength, and blood lactate regulation through enhanced AMPK/SIRT1 and PGC-1α signaling.

Rashet et al. (33) also demonstrated synergistic upregulation of AMPK/PGC-1α/SIRT1 signaling in the hippocampus of Alzheimer’s disease (AD) rats treated with aerobic treadmill exercise (6–18 m/min, 5 days/week for 8 weeks) and resveratrol (20 mg/kg/day), resulting in improved mitochondrial resilience and neuroprotection. These synergistic effects likely arise from complementary mechanisms: bioactives enhance AMPK phosphorylation via LKB1 and SIRT1 activation, whereas exercise amplifies ATP depletion and calcium signaling through CaMKK2, converging on shared metabolic and anti-inflammatory pathways.

However, many studies report additive rather than synergistic effects due to the absence of rigorous statistical interaction analyses. Lambert et al. (27) found that grape polyphenols and endurance treadmill training (up to 60 minutes per session at moderate intensity, 5 days/week for 12 weeks) in obese, insulin-resistant rats increased insulin sensitivity and endurance capacity by 68%, with stronger AMPK activation in the combined group compared to either intervention alone. Without interaction tests, these effects are classified as additive, reflecting parallel activation of AMPK/SIRT1/PGC-1α/Nrf2 pathways. Similarly, Yang et al. (29) demonstrated that DHA supplementation (15% of dietary lipids) combined with long-term aerobic treadmill exercise (3–12 m/min, 30 min/session, 3–5 days/week from 6 to 18 months of age) in aged obese mice enhanced hepatic AMPK phosphorylation, increased carnitine palmitoyltransferase 1a (CPT1A) and peroxisome proliferator-activated receptor alpha (PPARα) expression, and reduced inflammatory markers including monocyte chemoattractant protein 1 (MCP-1), IL-6, TNF-α, and Toll-like receptor 4 (TLR4). The absence of interaction testing suggests additive effects through overlapping metabolic and inflammatory mechanisms.

Dehghan et al. (38) reported that saffron (40 mg/kg/day) and resistance training in diabetic rats improved glucose uptake via AMPKα/glucose transporter 4 (GLUT4) signaling. The exercise protocol involved ladder climbing with incremental loads, three sessions per week for six weeks. While both interventions enhanced metabolic outcomes, the lack of factorial analyses precludes confirmation of synergistic effects. Liao et al. (31) also found that resveratrol (150 mg/kg/day) combined with aerobic treadmill running (20 m/min, 60 min/day, 5 days/week for 6 weeks) in elderly rats enhanced skeletal muscle AMPK/SIRT1 signaling, grip strength, and sarcomere integrity while reducing apoptosis. However, these effects were interpreted as additive rather than synergistic due to the absence of interaction analysis.

The distinction between synergistic and additive effects has significant implications for therapeutic design. Synergistic interactions, as seen in Kan et al. (30) and Rashet et al. (33), suggest that combined interventions may achieve greater-than-additive benefits, potentially reducing required doses or exercise intensity for clinical efficacy. Additive effects, as observed in Lambert et al. (27) and Yang et al. (29), indicate that bioactives and exercise enhance outcomes through parallel pathways, offering flexibility in combining interventions but requiring higher doses or intensities to achieve comparable effects. The variability in outcomes underscores the need for standardized protocols and interaction analyses to clarify the nature of these effects.

## Tissue-specific immunometabolic outcomes

**Figure 2:**
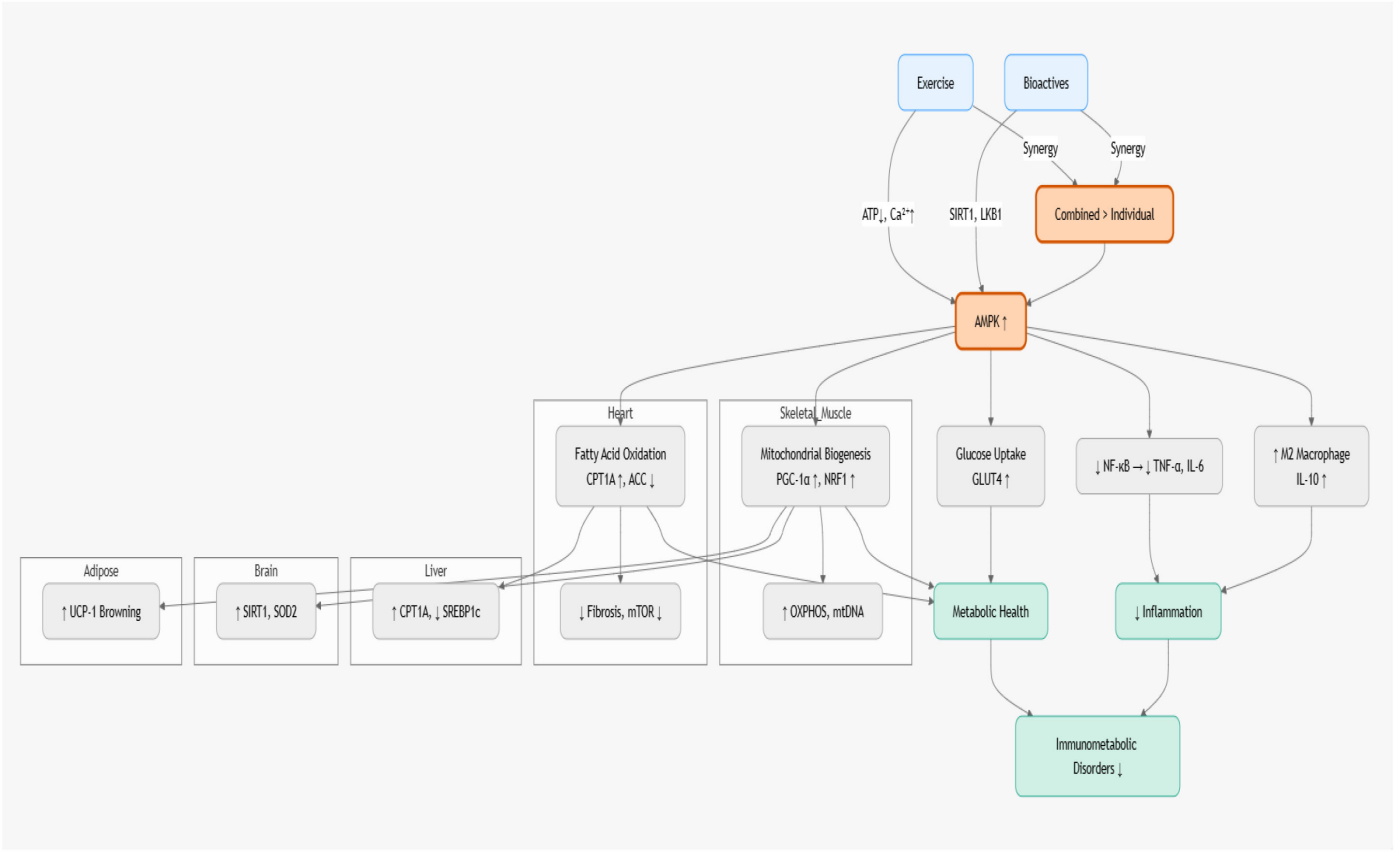
Exercise + bioactive compounds (quercetin, resveratrol, grape polyphenols, DHA, saffron) converge on AMPK via multiple upstream signals (ATP depletion, Ca²^+^/CaMKKβ, LKB1, SIRT1). Supra-additive synergy amplifies mitochondrial biogenesis, lipolysis, glucose uptake, and anti-inflammatory pathways—driving superior metabolic, cardiac, muscular, and neuroprotective outcomes compared to additive effects alone.

The studies reveal tissue-specific immunometabolic benefits, demonstrating the versatility of combined interventions across diverse physiological contexts (**Figure 2**). In skeletal muscle, Hamidie et al. (36) showed that curcumin supplementation (50–100 mg/kg/day, intraperitoneally for 28 days) combined with endurance treadmill running (60 min/day, 5 days/week for 4 weeks) in Wistar rats significantly enhanced mitochondrial biogenesis markers, including oxidative phosphorylation (OXPHOS) subunits, cytochrome c oxidase subunit IV (COX-IV), mitochondrial DNA (mtDNA) copy number, and citrate synthase (CS) activity. These effects were mediated via activation of AMPK/SIRT1/PGC-1α signaling and increased cyclic AMP (cAMP)–protein kinase A (PKA)–CREB phosphorylation, suggesting cross-talk between cAMP-dependent and AMPK-dependent pathways. While the study demonstrated strong molecular outcomes, its reliance on short-term intervention and absence of interaction statistics limit definitive conclusions on synergy versus additivity. Peyravi et al. (39) further demonstrated that crocin supplementation (25 mg/kg/day) combined with either HIIT (2 min at 85–90% VO__2__max alternating with 2 min at 50–60% VO__2__max, 5 sessions/week) or low-intensity continuous training (LICT; 60 min at 50–60% VO__2__max, 5 sessions/week for 8 weeks) in diabetic rat soleus muscle restored AMPK and nuclear respiratory factor 1 (NRF1) gene expression. This combined intervention enhanced mitochondrial function, oxidative metabolism, and glucose handling, underscoring the role of bioactive–exercise interactions in mitigating diabetes-related metabolic inflexibility. Liao et al. (31) found that resveratrol (150 mg/kg/day) combined with aerobic treadmill exercise (20 m/min, 60 min/day, 5 days/week for 6 weeks) in elderly rats increased skeletal muscle mass, grip strength, and AMPK/SIRT1 signaling while reducing apoptosis through decreased acetyl-p53 and Bax/Bcl-2 ratios, thereby attenuating sarcopenia.

**Figure 3:**
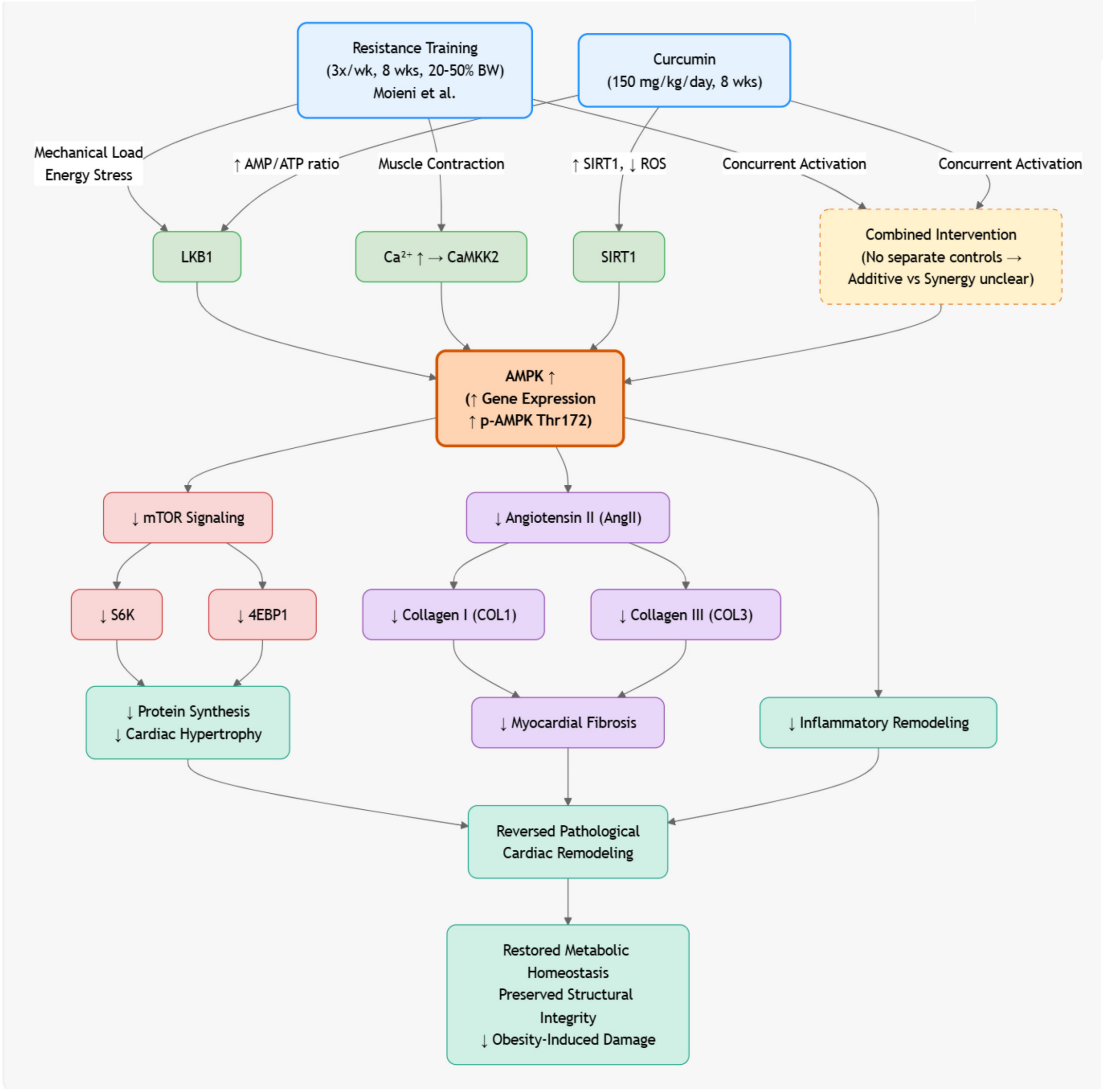
Exercise and bioactives (quercetin, resveratrol, grape polyphenols) activate AMPK, which suppresses mTORC1-mediated hypertrophy and fibrosis. This shifts cardiac metabolism toward fatty acid oxidation, upregulates PGC-1α-driven mitochondrial biogenesis, and restores autophagy—collectively reversing pathological remodeling, reducing interstitial collagen, and improving diastolic function in models of heart failure and metabolic stress.

In cardiac tissue (see **Figure 3**), Moieni et al. (37) demonstrated that resistance training combined with curcumin supplementation (150 mg/kg/day) in obese rats reversed pathological cardiac remodeling through coordinated modulation of AMPK and mammalian target of rapamycin (mTOR) signaling. The eight-week resistance training protocol (three sessions per week at 20–50% of body weight) significantly upregulated AMPK gene expression while downregulating mTOR, S6 kinase (S6K), eukaryotic translation initiation factor 4E-binding protein (4EBP), collagen type I (COL1), collagen type III (COL3), and angiotensin II (AngII). These changes attenuated myocardial fibrosis and inflammatory remodeling associated with obesity-induced cardiac hypertrophy. The findings suggest that concurrent mechanical loading and polyphenolic activation of AMPK can restore cardiac metabolic homeostasis and structural integrity by suppressing hypertrophic and profibrotic signaling cascades. However, the absence of separate exercise-only and curcumin-only control groups limits conclusions regarding potential synergistic effects, underscoring the need for factorial designs in future studies to distinguish additive from interactive contributions.

In the brain, Amirazodi et al. (32) reported that resveratrol supplementation (10 mg/kg/day) combined with HIIT in aged male rats enhanced hippocampal energy metabolism and redox balance through modulation of AMPK and sirtuin signaling. The six-week swimming-based HIIT protocol, performed five days per week with alternating high- and low-intensity bouts, significantly increased the hippocampal NAD^+^/NADH ratio, AMPK phosphorylation, and superoxide dismutase 2 (SOD2) expression. Notably, divergent sirtuin responses were observed—HIIT upregulated SIRT3, whereas resveratrol selectively increased SIRT4 expression—suggesting distinct yet complementary mitochondrial adaptations. The combined intervention enhanced neuroprotection and oxidative resilience in aged rats, supporting the premise that exercise-induced mitochondrial stress and resveratrol-mediated redox modulation converge on AMPK-dependent pathways to maintain neuronal homeostasis. However, the absence of statistical interaction analyses limits definitive conclusions about synergistic versus additive effects, highlighting the need for mechanistic studies with factorial designs. Ryu et al. (28) demonstrated that trans-cinnamaldehyde (TCA) supplementation combined with aerobic treadmill exercise effectively mitigated neuroinflammation and cognitive decline in a mouse model of mild cognitive impairment (MCI) induced by d-galactose and aluminum chloride. The eight-week treadmill protocol (five sessions per week, progressively increasing from 10 to 18 m/min for 30–60 min) and TCA administration (at a physiologically active dose) synergistically enhanced antioxidant defense via upregulation of nuclear factor Nrf2 and its downstream targets NAD(P)H quinone dehydrogenase 1 (NQO-1), heme oxygenase 1 (HO-1), and superoxide dismutase 1 (SOD1). These molecular adaptations were accompanied by reduced markers of neuroinflammation and improved performance in behavioral tests assessing learning and memory. The findings suggest that TCA and exercise act through convergent Nrf2-mediated redox signaling to enhance neuronal resilience and cognitive function. Nonetheless, the absence of formal interaction analysis precludes confirmation of true synergy, indicating additive effects through parallel activation of antioxidant pathways. Rashet et al. (33) demonstrated that aerobic treadmill training combined with resveratrol (20 mg/kg/day) supplementation synergistically upregulated hippocampal AMPK/PGC-1α/SIRT1 signaling in rats with AD, thereby enhancing mitochondrial resilience and neuroprotection. In this eight-week intervention, the aerobic exercise protocol involved treadmill running at incremental speeds ranging from 6 to 18 m/min, five days per week. Both interventions independently increased AMPK, PGC-1α, and SIRT1 expression compared with sedentary AD rats; however, the combined training-resveratrol group exhibited significantly greater increases in all three markers (P < 0.05), confirming a synergistic effect via two-way ANOVA. These findings suggest that resveratrol enhances AMPK phosphorylation through LKB1 and SIRT1 activation, while exercise amplifies ATP depletion and calcium-mediated CaMKK2 signaling, converging on shared metabolic and neuroprotective pathways. The study provides strong evidence that concurrent aerobic exercise and polyphenol supplementation can more effectively restore mitochondrial and cognitive function in AD than either intervention alone.

In adipose tissue, Mostafavian et al. (35) demonstrated that capsaicin (4 mg/kg/day) supplementation combined with eight weeks of progressive aerobic treadmill training significantly enhanced the expression of peroxisome proliferator-activated receptor gamma coactivator 1-alpha (PGC-1α) and uncoupling protein 1 (UCP-1) in the visceral adipose tissue (VAT) of high-fat diet (HFD) rats, promoting adipose tissue browning and improving metabolic flexibility. The aerobic training protocol consisted of progressive treadmill running for eight weeks, with incremental increases in duration and intensity to maintain overload and adaptation. Both capsaicin and exercise independently increased PGC-1α and UCP-1 gene expression relative to HFD controls; however, the combined capsaicin-plus-training group exhibited the most pronounced effects, surpassing the exercise-only and supplement-only groups (P < 0.05). These findings indicate that concurrent aerobic exercise and capsaicin supplementation synergistically activate thermogenic and mitochondrial biogenic pathways, mitigating obesity-induced insulin resistance through enhanced oxidative metabolism and energy expenditure. Medina-Contreras et al. (34) reported that topical capsaicin application (0.6 mg/kg/day; 0.075% cream) combined with moderate treadmill exercise in ovariectomized, hypoestrogenic obese rats synergistically reduced visceral adiposity, insulin resistance, and oxidative stress while enhancing skeletal muscle AMPK activation. The exercise protocol consisted of daily treadmill running for 20 minutes at speeds progressively increased from 9 to 18 m/min every 10 days over a six-week intervention period, with topical capsaicin applied 90 minutes prior to exercise in the combination group. Compared with individual treatments, the combined capsaicin-plus-exercise regimen (Cap+Ex) produced greater reductions in body weight, abdominal fat accumulation, and pancreatic islet hypertrophy, along with the highest increase in phosphorylated AMPK (p-AMPK) expression in the soleus muscle. These findings highlight the metabolic and anti-inflammatory synergy of moderate aerobic exercise and capsaicin in mitigating obesity- and estrogen-deficiency–related insulin resistance through AMPK-mediated improvements in glucose homeostasis and redox balance.

In the liver, Yang et al. (29) found that DHA supplementation and aerobic treadmill exercise in aged obese female C57BL/6J mice synergistically improved hepatic lipid metabolism and reduced inflammatory and stress-related signaling. In this study, after four months of high-fat diet feeding, diet-induced obese mice underwent a long-term intervention until 18 months of age, consisting of a DHA-enriched diet (15% of dietary lipids replaced by a DHA-rich concentrate) and/or treadmill running as the aerobic exercise protocol. The combination of DHA and exercise increased AMPK phosphorylation and upregulated CPT1A and PPARα expression, enhancing fatty acid oxidation. Concurrently, endoplasmic reticulum stress markers such as X-box binding protein 1 (Xbp1) and lipogenic genes (Dgat2, Scd1, Srebp1c) were suppressed, while autophagy-related proteins (p62, Atg7) were upregulated. Moreover, exercise, either alone or combined with DHA, significantly reduced hepatic inflammation by downregulating MCP-1, IL-6, TNF-α, and TLR4. Collectively, these findings indicate that long-term DHA supplementation and aerobic exercise cooperatively activate AMPK signaling, alleviate ER stress, and promote autophagic and oxidative pathways, mitigating NAFLD progression and hepatic inflammation during aging. These tissue-specific outcomes highlight the broad applicability of combined interventions, addressing diverse immunometabolic dysfunctions through AMPK-mediated pathways.

## Immunometabolic mechanisms

From an immunometabolic perspective, these interventions modulate critical pathways that integrate metabolic and immune responses. AMPK activation enhances mitochondrial biogenesis (via PGC-1α/NRF1), glucose uptake (GLUT4), and lipid oxidation (CPT1A, PPARα), while suppressing pro-inflammatory cytokines (TNF-α, IL-6) and oxidative stress (via Nrf2/HO-1/SOD). Bioactives like curcumin, resveratrol, capsaicin, crocin, and saffron reduce nuclear factor-kappa B (NF-κB) signaling and promote anti-inflammatory M2 macrophage polarization, as evidenced by Medina-Contreras et al. (34) and Moieni et al. (37). For instance, curcumin’s inhibition of NF-κB and promotion of M2 macrophages in obese rat hearts reduced fibrosis and inflammation, countering obesity-induced immune-metabolic dysregulation. Similarly, capsaicin’s effects in ovariectomized rats (34) involved M2 polarization and reduced oxidative stress, supporting insulin sensitivity.

Exercise, particularly HIIT and resistance training, enhances myokine production (e.g., muscle-derived IL-6) and insulin sensitivity, as shown by Lambert et al. (27) and Dehghan et al. (38). HIIT and resistance training also increase cAMP and PKA signaling, activating CREB and LKB1, which amplify AMPK and anti-inflammatory effects, as seen in Hamidie et al. (2015). These interventions collectively improve metabolic flexibility by enhancing substrate utilization (e.g., glycogen sparing, lipid oxidation) and reducing chronic low-grade inflammation, a hallmark of immunometabolic disorders. For example, Mostafavian et al. (2020) demonstrated that capsaicin and exercise-induced UCP-1 overexpression in adipose tissue promotes thermogenesis and reduces inflammation, countering obesity-related insulin resistance. In the brain, Rashet et al. (33) and Ryu et al. (33) showed that AMPK/SIRT1/Nrf2 signaling mitigates neuroinflammation and oxidative stress, critical for AD and MCI. Dehghan et al. (38) highlighted saffron’s role in enhancing AMPKα/GLUT4 signaling in diabetic rats, improving glucose uptake and reducing oxidative stress-mediated insulin resistance.

AMPK modulates immune-metabolic interactions by balancing pro- and anti-inflammatory responses. The convergence of bioactives and exercise on pathways like SIRT1/PGC-1α/Nrf2 and NF-κB underscores their potential to reprogram systemic immunometabolism, enhancing tissue resilience and metabolic efficiency.

## Immunological mechanisms of combined interventions

Combined bioactive–exercise interventions modulate immunometabolic homeostasis primarily through AMPK-mediated suppression of NF-κB signaling, leading to reduced production of pro-inflammatory cytokines such as TNF-α and IL-6 and promoting a shift toward M2 macrophage polarization. For instance, curcumin, combined with resistance training, downregulated cardiac NF-κB and enhanced M2-associated markers, including IL-10 and arginase-1, thereby mitigating fibrosis and inflammation in obese rats (37). Similarly, capsaicin administration, combined with moderate exercise, in ovariectomized rats increased M2 macrophage polarization and reduced insulin resistance (34). These effects are closely linked to the activation of the SIRT1 and Nrf2 pathways, which enhance antioxidant defenses and suppress the transcription of inflammatory genes.

At the molecular level, AMPK activation integrates metabolic and redox signaling by phosphorylating downstream targets such as PGC-1α and FOXO transcription factors, promoting mitochondrial biogenesis and autophagy (74–77). Moreover, emerging evidence suggests that epigenetic regulation plays a critical role in sustaining these adaptive responses. For example, AMPK–SIRT1 signaling can induce histone deacetylation (e.g., reduced H3K9ac and H3K27ac) and regulate microRNAs such as miR-33, miR-34a, and miR-146a, which in turn modulate genes involved in lipid oxidation, inflammation, and macrophage phenotype switching (78–81). These epigenetic modifications may act as long-term “metabolic memory” mechanisms, stabilizing anti-inflammatory and oxidative stress–resistant phenotypes even after intervention cessation. Understanding the AMPK/SIRT1/PGC-1α axis is of utmost importance as it defines how bioactives and exercise jointly reprogram cellular metabolism and immunity, and it is a significant area of research in the field.

## Methodological strengths, limitations, and translational relevance of preclinical studies

A more precise comparison of the strengths and weaknesses of the above studies is necessary to contextualize their translational relevance. Collectively, these preclinical investigations demonstrate consistent activation of AMPK/SIRT1/PGC-1α signaling and downstream metabolic or anti-inflammatory pathways across diverse tissues, supporting the mechanistic plausibility of combining bioactives with exercise. This promising approach, with its key strength lying in its controlled experimental designs, inclusion of multiple intervention arms (exercise alone, bioactive alone, and combined), and use of molecular markers to confirm pathway activation, offers a hopeful path for future research. Moreover, several studies, such as those by Kan et al. (30) and Rashet et al. (33), employed two-way ANOVA to test for interaction effects, providing more substantial evidence for true synergy. However, most reports are limited by small sample sizes, lack of standardized dosing regimens, and heterogeneous exercise protocols (ranging from HIIT to moderate treadmill running or resistance training), which complicate cross-study comparisons. Many studies also lacked long-term follow-up to assess the durability of effects or failed to include statistical interaction analyses, leading to cautious classification of outcomes as additive rather than synergistic. The variability in animal models—spanning obesity, diabetes, aging, and neurodegenerative disease—further challenges extrapolation to human physiology.

Despite promising results, several methodological limitations persist. Most studies, including those by Ryu et al. (33), Yang et al. (29), Peyravi et al. (39), and Dehghan et al. (38), did not employ rigorous statistical tests (e.g., two-way ANOVA with interaction terms) to confirm synergistic effects, necessitating cautious interpretation. Considerable variability in bioactive dosages (e.g., resveratrol 10–150 mg/kg/day, curcumin 50–150 mg/kg/day, capsaicin 0.6–4 mg/kg/day) and exercise duration or intensity (e.g., HIIT vs. MICT, 6–28 weeks) further limits reproducibility and comparability. For instance, resveratrol’s effects ranged from 10 mg/kg/day (32) to 150 mg/kg/day (31), producing inconsistent outcomes. The absence of single-treatment control groups in some studies, such as Moieni et al. (37), hinders the ability to attribute observed benefits to either bioactives, exercise, or their combination. These limitations underscore the need for further investigation and improvement in the field.

It is important to note that none of the reviewed studies explored epigenetic mechanisms (e.g., histone acetylation, DNA methylation, or microRNA regulation) that could mediate sustained AMPK activation and immunometabolic reprogramming. This represents a critical research gap and underscores the potential for further investigation in this area. Furthermore, comprehensive immunological profiling was often lacking; few studies assessed cytokine arrays or immune cell phenotypes (e.g., Tregs, M1/M2 macrophage polarization), limiting understanding of systemic immunometabolic effects. Finally, all current evidence remains preclinical, and translational barriers—including interspecies differences in AMPK signaling and dose-scaling challenges—underscore the urgent need for well-designed human trials to validate and refine these combined interventions.

Ultimately, although these studies offer valuable mechanistic insights, they fail to adequately bridge the translational gap between animal models and clinical applications. Significant differences in bioactive metabolism, dosing scalability, and exercise adaptation limit direct extrapolation to humans. Addressing these challenges—through the implementation of standardized preclinical protocols, rigorous dose–response analyses, and the initiation of well-designed human clinical trials—would significantly enhance the translational relevance and therapeutic potential of combined bioactive–exercise interventions. The urgency and importance of these actions cannot be overstated.

## Clinical implementation, safety, and human translation

Implementing combined bioactive and exercise protocols in clinical practice will require more precise guidance than is currently available. One issue that keeps resurfacing is the issue of dosing. The quantities used in animal experiments—resveratrol in the range of 10–150 mg/kg/day or curcumin between 50–150 mg/kg/day—are far above what patients typically tolerate or even absorb. Human studies report that resveratrol above roughly 500 mg/day often leads to gastrointestinal symptoms (82). Curcumin presents a different challenge, as most formulations are poorly absorbed unless coupled with an appropriate carrier (83), and capsaicin must be administered in amounts that provide metabolic benefits without causing excessive sensory irritation (84). Clinical protocols, as a result, need to rely more heavily on careful pharmacokinetic evaluation, standardized delivery systems such as particulate curcumin or coated capsaicin capsules, and slow dose escalation.

Exercise prescriptions raise another set of questions. Rodent work draws on HIIT programs, conventional treadmill sessions, or resistance training; however, none of these can be directly transferred to clinical settings without adjustment. Age, existing medical conditions, and individual fitness determine what patients can safely perform. Moderate continuous exercise is generally more realistic for individuals with obesity or early-stage type 2 diabetes (85). In contrast, high-intensity intervals tend to require closer supervision and a more stable metabolic profile (86). Clinicians also need to monitor for events associated with glucose fluctuations, cardiovascular strain, or interactions with medications used to control blood pressure, glucose, or lipids.

Evidence of a measurable benefit in humans remains thin. Some early trials report modest gains in insulin sensitivity, indices of mitochondrial activity, or inflammatory markers when bioactives such as resveratrol or curcumin are used, but confirmation of any combined effect with exercise is lacking. Studies that use factorial designs—separating bioactive, exercise, and combined arms—and include biomarkers tied to AMPK, SIRT1, or PGC-1α signaling are needed to clarify the picture. Any eventual clinical use will hinge on results that clearly surpass the impact of exercise on its own, together with dosing plans that account for interindividual differences in genetic and epigenetic features relevant to metabolic regulation.

## Clinical implications and future directions

Activation of the AMPK/SIRT1/PGC-1α axis through combined bioactive supplementation and exercise represents a promising framework for personalized metabolic disease management. In individuals with insulin resistance or early-stage type 2 diabetes, tailored interventions integrating endurance training with resveratrol or curcumin may enhance mitochondrial biogenesis, improve glucose utilization, and optimize glycemic control. Likewise, in obese or postmenopausal populations with elevated inflammatory markers, capsaicin or DHA supplementation combined with moderate aerobic exercise could promote adipose browning and attenuate systemic inflammation. Importantly, genetic and epigenetic variability—including polymorphisms or methylation differences in SIRT1, AMPK, or PGC-1α—may influence responsiveness to such interventions, supporting the advancement of precision exercise–nutrition strategies. Collectively, these findings highlight the translational potential of bioactive–exercise combinations as individualized therapies for metabolic and inflammatory disorders.

To address these gaps and advance the field, future research should prioritize the following:

Statistical Validation: Employ robust statistical methods, such as two-way ANOVA with interaction terms, to confirm synergistic interactions, addressing ambiguities in studies like Lambert et al. (27), Yang et al. (29), and Ryu et al. (33).

Epigenetic Mechanisms: Investigate how bioactives and exercise modulate epigenetic regulators (e.g., histone acetylation, miRNAs) to influence AMPK and inflammatory gene expression, aligning with the journal’s interest in transcriptional regulation.

Clinical Studies: Translate preclinical findings to human trials, optimizing bioactive dosages (e.g., standardized equivalents to preclinical doses) and exercise regimens (e.g., HIIT vs. endurance) for specific immunometabolic disorders, such as type 2 diabetes, NAFLD, or AD.

Tissue-Specific Mechanisms: Elucidate differential effects across tissues (e.g., adipose vs. brain) and their systemic interplay, using multi-omics approaches (e.g., transcriptomics, metabolomics) to map AMPK-mediated pathways and immune-metabolic interactions.

Cytokine and Immune Profiling: Assess changes in adipokines (e.g., adiponectin), cytokines (e.g., IL-6, TNF-α, IL-10), and macrophage polarization (M1 vs. M2) to comprehensively characterize immunometabolic responses, as suggested by Medina-Contreras et al. (34).

Standardized Protocols: Develop standardized bioactive dosages and exercise protocols to enhance reproducibility and facilitate meta-analyses, addressing variability in resveratrol (10–150 mg/kg/day), curcumin (50–150 mg/kg/day), and exercise durations (6–28 weeks).

Furthermore, future research should leverage integrative experimental models and molecular biomarkers to unravel AMPK-mediated immunological mechanisms. Tissue-specific knockout models for AMPKα1/α2, SIRT1, or PGC-1α can delineate causal roles in immunometabolic remodeling, while disease-specific animal models (e.g., high-fat diet–induced obesity, streptozotocin-induced diabetes, or APP/PS1 Alzheimer’s models) can evaluate physiological relevance. Biomarkers such as phospho-AMPK (Thr172), phospho-ACC (Ser79), and downstream effectors (PGC-1α, NRF1, CPT1A, UCP1) should be standardized as readouts of AMPK activation. Finally, the integration of omics-based data will enable discovery of novel miRNAs, histone modifications, and metabolic signatures, providing mechanistic insight into AMPK’s immunoregulatory functions. Together, these experimental and analytical frameworks will strengthen the mechanistic foundation and accelerate the clinical translation of combined bioactive–exercise interventions within precision medicine paradigms.

## Conclusion

Combining natural bioactives and physical activity potently modulates AMPK signaling, enhancing metabolic flexibility and suppressing chronic inflammation via NF-κB inhibition and M2 macrophage polarization. These findings position combined interventions as accessible, cost-effective therapies for immunometabolic disorders, including obesity, type 2 diabetes, and neurodegenerative conditions. Addressing limitations in statistical validation, protocol standardization, and clinical translation will enable personalized therapeutic strategies, leveraging immunological and epigenetic insights to improve global health outcomes.
